# Acute Decompensation Requiring Cardiopulmonary Resuscitation in a 15-Year-Old With Undiagnosed Lyme Carditis

**DOI:** 10.7759/cureus.110480

**Published:** 2026-06-08

**Authors:** Anne-Sophie Gervais, Joshua Feder, Earl Rubin, Virginie Beauséjour-Ladouceur, Marina Salvadori

**Affiliations:** 1 Pediatric Intensive Care Unit, Montreal Children's Hospital, McGill University, Montreal, CAN; 2 Infectious Diseases, Montreal Children's Hospital, McGill University, Montreal, CAN; 3 Cardiology, Montreal Children's Hospital, McGill University, Montreal, CAN

**Keywords:** complete atrioventricular block, lyme's disease, permanent pacemaker avoidance, subclinical carditis, sudden cardiac death (scd)

## Abstract

We describe the case of a previously healthy teenager who developed third-degree atrioventricular block due to Lyme disease. While Lyme carditis is common in children, severe conduction anomalies remain rare but can be life-threatening. This case highlights the importance of early recognition, prompt testing, and timely intervention to prevent unnecessary procedures, such as permanent pacemaker implantation, and to ensure favorable outcomes. This case also supports Lyme disease testing in pediatric sudden cardiac death in cases with autopsy findings suggestive of myocarditis or pericarditis.

## Introduction

Lyme disease, caused by *Borrelia burgdorferi* and transmitted by *Ixodes* ticks, is the most common vector-borne infection in North America and is increasing in incidence because of climate change [1]. Cardiac involvement, or Lyme carditis, is an early disseminated manifestation and most commonly presents as atrioventricular conduction abnormalities. These conduction disturbances can vary in severity, from first-degree atrioventricular block to complete heart block, and may fluctuate over a short period of time and occasionally require pacing [1,2]. We report the case of a previously healthy teenager who presented with acute decompensation requiring cardiopulmonary resuscitation due to undiagnosed Lyme carditis.

## Case presentation

A 15-year-old male presented to an outpatient clinic on the South Shore of Montreal, Quebec, in November, with a two-day history of fatigue. He was found to be in complete heart block at triage with a ventricular rate of around 50 bpm. After a blood draw, the patient had a syncopal episode, further bradycardia into the 20s, and received cardiopulmonary resuscitation (CPR) for approximately 20 seconds. He regained consciousness but had persistent bradycardia with a heart rate as low as 25 bpm. He was promptly transferred to a tertiary care center in Montreal for further management.

On arrival to the emergency department, the patient had complete heart block with ventricular escape rhythm between 30 and 55 bpm (Figure 1) and a systolic blood pressure as low as 60 mmHg. He received epinephrine boluses to maintain a heart rate above 35 bpm and a normal blood pressure. His saturation and respiratory rate were within normal limits. He was afebrile. He was alert and oriented and showed no neurological deficits. He was admitted to the intensive care unit (ICU) and was connected to the defibrillator for potential transcutaneous pacing but never required it. He subsequently had three symptomatic bradycardic episodes related to minor efforts, with heart rate as low as 20 bpm and hypotension, which resolved without intervention. These episodes prompted the installation of a transjugular temporary pacemaker, initially at a VVI (ventricle paced, ventricle sensed, inhibited) rate of 60 bpm, to prevent symptomatic bradycardia. The patient was empirically started on ceftriaxone (2 grams IV once daily) on admission to treat possible Lyme carditis, as the south shore of Montreal is an endemic region for Lyme.

**Figure 1:**
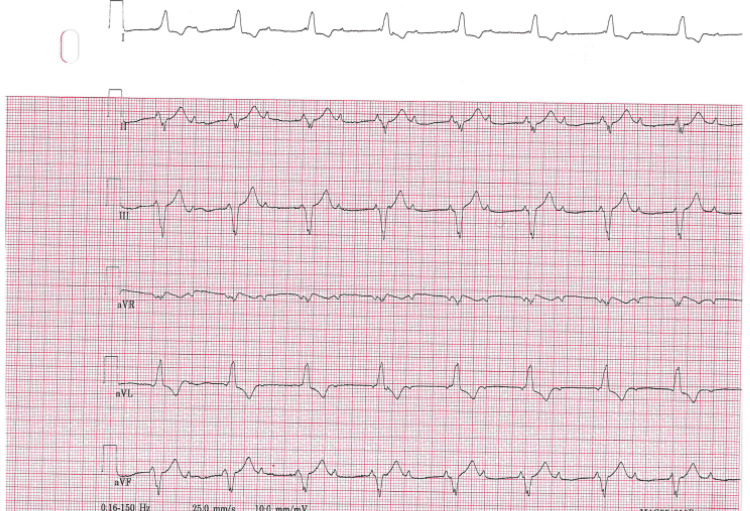
Rhythm strips on arrival to the tertiary hospital Complete atrioventricular block with wide complex escape rhythm at 50 bpm and atrial rate at 110

The patient had no recent viral illness or fever. He had only travelled to Boston and New York in urban areas within the last year and had no known tick bites. He had a history of vasovagal episodes related to noxious stimuli but was otherwise previously healthy. He played soccer almost daily in the fields near his house, where the grass was not always closely cut, wearing shoes and short socks that extended only to his lower ankles. There was no family history of arrhythmia, sudden death, or pacemaker use.

The echocardiogram showed normal biventricular function and normal cardiac anatomy (Video 1), and his chest X-ray was within normal limits (Figure 2). Complete blood count and coagulation studies were within normal limits. His peak lactate was 8.3 mmol/L, which normalized within 12 hours after inotropic support initiation. ALT was slightly elevated, but the rest of his liver panel was unremarkable. His creatinine and urea were slightly elevated. His venous and arterial gases showed no acidosis, and his initial electrolytes were within normal limits. His CRP was 17 mg/L, and procalcitonin was 0.117 ug/L. His initial pro-BNP was 871 pg/mL and troponin was 9.4 ng/L. His nasopharyngeal swab was negative for viruses. His autoimmune serologies (anti-DNA, ANA, anti-MPO, anti-ENA) were negative. Lyme serology was sent on admission and performed at the Quebec Public Health Laboratory (LSPQ) using a two-step ELISA-based algorithm. The initial IgG/IgM screening test with a ZEUS ELISA detecting antibodies against VlsE1 and pepC10 antigens of *B. burgdorferi*, including *B. burgdorferi, B. afzelii*, and *B. garinii*, was positive. Confirmatory IgG testing by ZEUS ELISA was also positive, consistent with confirmed Lyme disease. Laboratory findings are detailed in Table 1, and serological findings are detailed in Table 2.

**Video 1:** Echocardiogram on presentation Normal cardiac anatomy. Grossly normal left ventricular systolic function. Grossly normal right ventricular systolic function. These findings were reassuring, demonstrating preserved biventricular function and no structural cardiac abnormalities despite markedly abnormal cardiac biomarkers and advanced atrioventricular block. This is consistent with Lyme carditis predominantly affecting the conduction system rather than causing extensive myocardial dysfunction.

**Figure 2:**
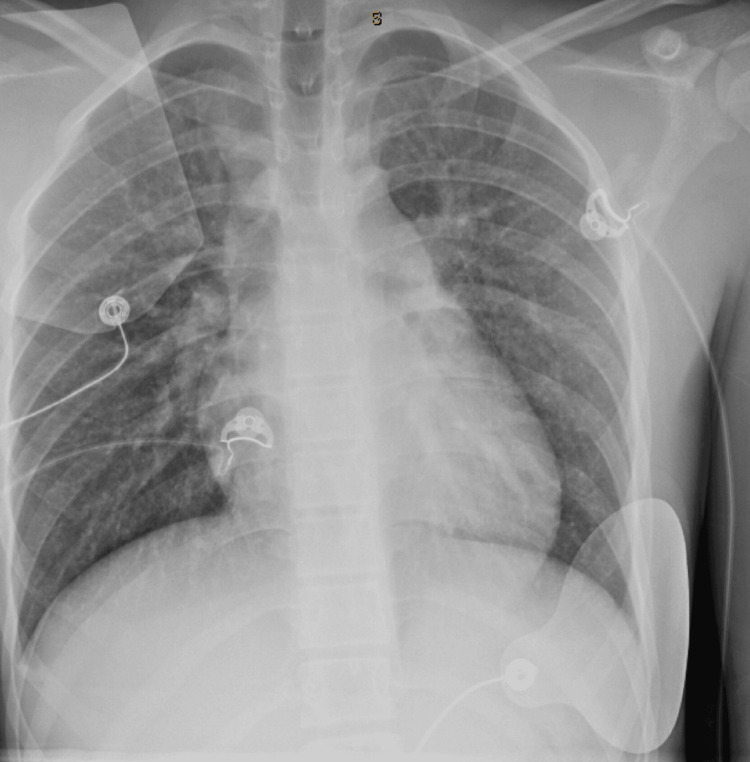
Chest X-ray on presentation The lungs show no focal opacity or consolidation and no significant pleural effusion. The cardiothymic silhouette is unremarkable.

**Table 1:** Laboratory findings on presentation Increased lactate in the context of reduced cardiac output due to severe bradycardia and complete heart block, leading to systemic hypoperfusion. Increased BNP in the context of cardiac stress related to Lyme carditis. INR: international normalized ratio, PT: prothrombin time, PTT: partial thromboplastin time, ALT: alanine aminotransferase, GGT: gamma-glutamyl transferase, BNP: B-type natriuretic peptide

Laboratory tests	Patient values	Reference range	Units
White count	11.60	4.50-13.00	10^9^/L
Hemoglobin	138	115-155	g/L
Platelets	327	140-450	10^9^/L
Venous pH	7.36	7.34-7.44	
CO_2_	33.5	41-51	mmHg
Bicarbonate	18.6	21-26	mmol/L
Lactate	8.3	0.6-2.4	mmol/L
Sodium	138	137-144	mmol/L
Potassium	3.2	3.0-5.6	mmol/L
Chloride	107	99-110	mmol/L
INR	1.11	0.88-1.12	
PT	14.9	12.4-15.0	s
PTT	32.9	29.1-42.2	s
Procalcitonin	0.117	<= 0.065	ug/l
C-reactive Protein	17.80	0-5	mg/L
Urea	4.8	2.0-6.8	mmol/L
Creatinine	96	38-75	umol/L
ALT	110	0-18	U/L
Alkaline phosphatase	105	77-317	U/L
GGT	83	0-40	U/L
Troponin I	9.4	<=17.5	ng/L
BNP	871	NA	picograms/mL

**Table 2:** Serological findings Negative workup from an autoimmune perspective.

Serological tests	Patient’s values	Reference range	Units
Anti-SSA (RO)	<2	0-19	RU/ml
Anti-SSB (LA)	<2	0-19	RU/ml
Anti-Smith	<2	0-19	RU/ml
Anti-RNP	<2	0-19	RU/ml
ANA Titre	1:80	NA	NA
Anti-PR3	<2	0-19	RU/ml
Anti-MPO	<2	0-19	RU/ml

The patient had a stress test conducted on the 11th day of admission after showing signs of recovery and requiring infrequent pacing. At the time, his baseline ECG was a normal sinus rhythm with first-degree atrioventricular block and some inferior T-wave inversion (Figure 3). There was 1:1 conduction throughout exercise and recovery up to a maximal heart rate of 150 bpm. No higher-grade atrioventricular block was seen, and normal blood pressure was maintained throughout the test. There were no signs of ischemia or arrhythmia. These reassuring findings demonstrated adequate conduction reserve and supported safe discharge with the temporary pacemaker left in situ.

**Figure 3:**
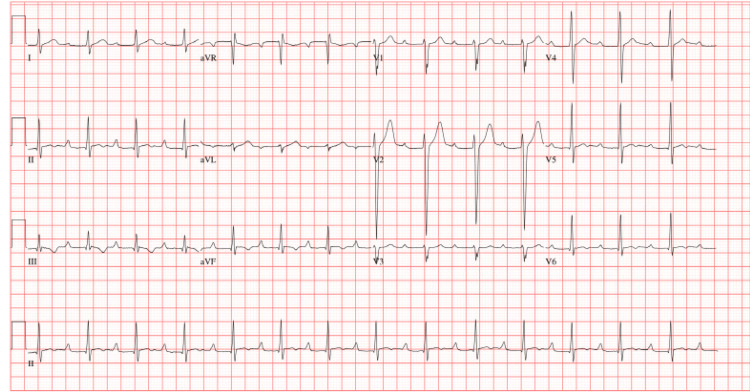
Baseline ECG Sinus rhythm with first-degree block (PR 294 ms). T-wave inversion in inferior leads.

The patient was discharged home from the pediatric intensive care unit (PICU) on the 11th day of hospitalization to complete a total of 21 days of ceftriaxone as an outpatient. He was discharged with the transjugular temporary pacemaker in situ. This was removed as an outpatient, 11 days after discharge, due to normalization of his ECG and acceptable follow-up stress test results. One month following discharge, his electrocardiogram and Holter showed a normal PR interval and no signs of higher-grade atrioventricular block (Figure 4). At follow-up, the patient had no neurological sequelae and had returned to his baseline level of functioning. Figure 5 describes the timeline of the case. 

**Figure 4:**
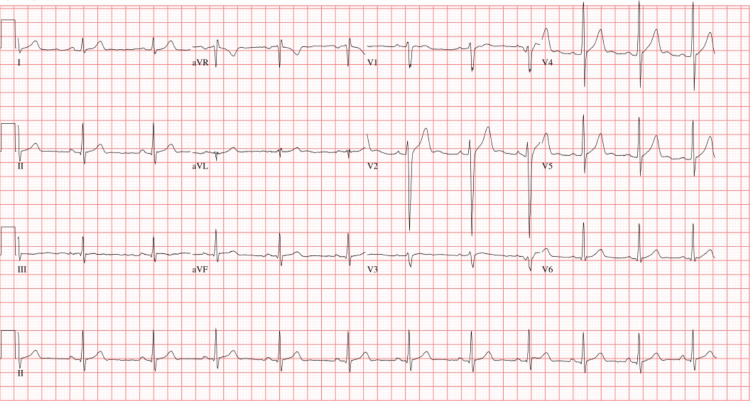
Follow-up ECG one month after discharge Normal ECG, PR segment 154 ms

**Figure 5:**
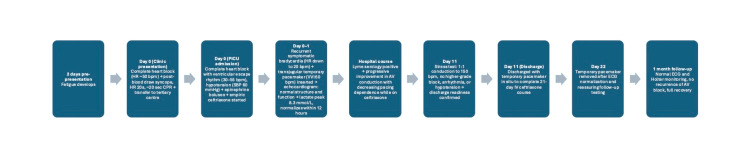
Timeline of the case

## Discussion

Lyme carditis occurs in approximately 4-10% of untreated adults with Lyme disease, with a peak incidence in childhood and middle age. The hallmark presentation of Lyme carditis is atrioventricular nodal block, often with rapidly progressive complete heart block. This condition typically develops within several days to a month following the initial infection and during the summer and fall seasons [1]. Pediatric-specific data reveal that 16% of children with early disseminated Lyme disease develop carditis, with 42% of those cases presenting with second-degree or higher-grade heart block, including 27% with complete heart block. The spectrum of disease in Lyme carditis is wide, from asymptomatic first-degree block to fulminant myocarditis requiring a higher level of support, such as mechanical ventilation, temporary pacing, and inotropes. In one study, four children (12%) had decreased ventricular systolic function, four children (12%) required temporary transvenous pacing, and one patient required cardiopulmonary resuscitation and extracorporeal membrane oxygenation (ECMO) [2]. However, permanent pacemaker implantation is rarely needed since recovery with supportive care and antibiotic treatment is the norm, usually within three to seven days [1-3]. Furthermore, the majority of patients with previous advanced or complete heart block had normalization of their PR interval on follow-up [2,3]. Therefore, Lyme testing should be performed prior to pacemaker implantation for pediatric patients with heart block of unknown cause in endemic areas, as identifying Lyme carditis would prevent unnecessary permanent pacemaker implantation in otherwise healthy young individuals.

Despite the excellent treatment prognosis for Lyme carditis, mortalities have been reported, both in adulthood and childhood [4-7]. One case study reported the death of a 17-year-old male with a three-week history of a viral prodrome who was found unresponsive on his lawn [4]. He was resuscitated and stabilized, but rapidly deteriorated and progressed from ventricular fibrillation to pulseless electrical activity to asystole. His autopsy showed cardiomegaly, myocardial interstitial fibrosis, and a Warthin-Starry stain revealed spirochetes in the ventricular myocardium. ELISA was positive for *B. burgdorferi*, which was subsequently confirmed by Western blot testing and polymerase chain reaction (PCR) in the myocardium, lung, and brain. A similar case of a 37-year-old male was reported [5]. He presented with non-specific upper respiratory tract symptoms with intermittent fevers for two weeks, one month before his death, then developed a diffuse annular rash, and presented to an emergency setting with second-degree atrioventricular block. The next morning, the patient was found unresponsive in his home. His autopsy showed a moderately enlarged heart, with histology showing severe and diffuse carditis. Serologic testing done the day before the patient died was positive (ELISA) for *B. burgdorferi* with a positive confirmatory Western blot. PCR for *B. burgdorferi* was positive. 

In these two cases, the patients were both tested for Lyme disease due to living in an endemic region or epidemiologic risk factors. However, neither patient had a clear history of a tick bite. An epidemiologic study showed that approximately 40% of patients diagnosed with Lyme carditis have a history of characteristic erythema migrans skin rash [8], and patients often only have nonspecific symptoms, which increases the risk of not recognizing Lyme disease, and therefore, of having cases of sudden death from cardiac involvement. Furthermore, half of pediatric patients with electrocardiographic evidence of Lyme carditis have no cardiac symptoms [2]. In our case, the patient had two days of fatigue and no history of rash or tick bite, making the diagnosis extremely challenging if not for the acute presentation of complete heart block. With climate change, the prevalence of Lyme disease is increasing, and clinicians are expected to see more Lyme carditis, both in endemic and previously non-endemic regions [9], increasing the potential for fatal cases like those described above.

The Infectious Diseases Society of America (IDSA) 2020 Guidelines for the Prevention, Diagnosis, and Treatment of Lyme Disease recommend testing for Lyme disease in all patients with acute myocarditis or pericarditis of unknown cause in an appropriate epidemiologic setting [1]. Lyme testing should also be performed prior to pacemaker implantation for young patients with heart block of unknown etiology, as Lyme carditis identification and treatment would prevent the need for such a procedure. Given the increasing incidence and geographic expansion of Lyme disease [9], consideration could also be given to incorporating Lyme disease testing into the autopsy evaluation of pediatric sudden cardiac death cases when the clinical history, epidemiologic context, or cardiac pathology raises suspicion for myocarditis or pericarditis. As there are no consensus diagnostic histologic criteria to date [4], further testing with tissue PCR and other stains should be performed for identification of Lyme carditis. Since antibiotic treatment can be lifesaving in acute cases, identifying Lyme carditis at autopsy is important for public health surveillance, as it may reveal unrecognized endemic areas.

## Conclusions

Our case highlights the risk of mortality from Lyme carditis if rapid management of the patient had not been provided, given the lack of overt cardiac symptoms prior to presentation that suggest Lyme. The increasing prevalence of Lyme disease and high percentage of high-grade AV block in pediatric cases should raise awareness for more potentially fatal cases of Lyme carditis and routine autopsy testing, particularly if signs of myopericarditis are present.
